# Climate drives the spatiotemporal dynamics of scrub typhus in China

**DOI:** 10.1111/gcb.16395

**Published:** 2022-09-02

**Authors:** Fangyu Ding, Qian Wang, Mengmeng Hao, Richard James Maude, Nicholas Philip John Day, Shengjie Lai, Shuai Chen, Liqun Fang, Tian Ma, Canjun Zheng, Dong Jiang

**Affiliations:** ^1^ Institute of Geographic Sciences and Natural Resources Research Chinese Academy of Sciences Beijing China; ^2^ College of Resources and Environment University of Chinese Academy of Sciences Beijing China; ^3^ Nuffield Department of Medicine, Centre for Tropical Medicine and Global Health University of Oxford Oxford UK; ^4^ Mahidol Oxford Tropical Medicine Research Unit, Faculty of Tropical Medicine Mahidol University Bangkok Thailand; ^5^ Harvard TH Chan School of Public Health Harvard University Boston Massachusetts USA; ^6^ WorldPop, School of Geography and Environmental Science University of Southampton Southampton UK; ^7^ State Key Laboratory of Pathogen and Biosecurity Beijing Institute of Microbiology and Epidemiology Beijing China; ^8^ Chinese Center for Disease Control and Prevention Beijing China

**Keywords:** climate change, impacts, public health, scrub typhus, spatiotemporal dynamic, vector‐borne disease

## Abstract

Scrub typhus is a climate‐sensitive and life‐threatening vector‐borne disease that poses a growing public health threat. Although the climate‐epidemic associations of many vector‐borne diseases have been studied for decades, the impacts of climate on scrub typhus remain poorly understood, especially in the context of global warming. Here we incorporate Chinese national surveillance data on scrub typhus from 2010 to 2019 into a climate‐driven generalized additive mixed model to explain the spatiotemporal dynamics of this disease and predict how it may be affected by climate change under various representative concentration pathways (RCPs) for three future time periods (the 2030s, 2050s, and 2080s). Our results demonstrate that temperature, precipitation, and relative humidity play key roles in driving the seasonal epidemic of scrub typhus in mainland China with a 2‐month lag. Our findings show that the change of projected spatiotemporal dynamics of scrub typhus will be heterogeneous and will depend on specific combinations of regional climate conditions in future climate scenarios. Our results contribute to a better understanding of spatiotemporal dynamics of scrub typhus, which can help public health authorities refine their prevention and control measures to reduce the risks resulting from climate change.

## Graphical abstract

By combining Chinese national surveillance data on scrub typhus from 2010 to 2019 with a climate‐driven generalized additive mixed model, this study explained the spatiotemporal dynamics of this disease and predicted how it may be affected by climate change under various representative concentration pathways for three future time periods. We found that the change of projected spatiotemporal dynamics of scrub typhus will be heterogeneous and will depend on specific combinations of regional climate conditions in future climate scenarios. This is valuable for helping public health authorities refine their prevention and control measures to reduce the risks resulting from climate change.

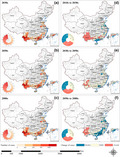

## 1 INTRODUCTION

The potential impacts of climate change on public health have become one of the most challenging environmental issues (Patz et al., 2005; Rocklöv & Dubrow, 2020; Rogers & Packer, 1993). In particular, the effects of climate change on spatiotemporal dynamic patterns of vector‐borne infectious diseases have garnered substantial research interest. Given that the viral and bacterial infectious agents and their associated vector organisms (e.g., mosquitoes, ticks, and mites) are largely devoid of thermostatic mechanisms, many aspects of their biology (i.e., reproduction and survival rates) are highly susceptible to changing climate (Caldwell et al., 2021; Franklinos et al., 2019; Iwamura et al., 2020). This fact, combined with the constant threat that vector‐borne diseases pose to public health and the economy in many regions (Kilpatrick & Randolph, 2012), renders it crucial to quantify the impacts of climate change on such diseases.

Scrub typhus is a life‐threatening vector‐borne disease caused by the bacterium *Orientia tsutsugamushi*, which is occasionally transmitted to humans during feeding of larval mites (Lim et al., 2020). The disease was traditionally thought to be restricted to a well‐defined geographic region called the “Asia‐Pacific tsutsugamushi triangle,” extending from far eastern Russia to northern Japan, northern Australia, the islands of the southwestern Pacific, and Afghanistan in the west (Kelly et al., 2009). It is estimated that scrub typhus potentially threatens more than 1 billion persons and causes 1 million clinical cases per year globally (Weitzel et al., 2016). The broad range of its clinical manifestations includes eschar, headache, fever, chills, rashes, enlarged lymph nodes, and mental changes (Cho et al., 2007; Paris & Day, 2020). Human infection may cause severe complications, including meningitis, meningoencephalitis, acute respiratory distress syndrome, myocarditis, multiple‐organ failure, and bleeding diatheses, all of which can be fatal if not appropriately treated (Dittrich et al., 2015; Olsen et al., 2015). In recent decades, under the situation where no effective vaccines are available against the disease, scrub typhus has been jeopardizing public health with the growing number of cases of human infection and the geographical range of infected areas (Walker, 2016; Zheng et al., 2018).

Previous studies have documented that the spatiotemporal dynamics of scrub typhus are sensitive to climate factors (Li et al., 2020; Mayxay et al., 2013). For instance, transmission in southern China and northern Japan is evidently seasonal, occurring primarily in summer (Liu et al., 2006; Seto et al., 2017). On the other hand, in northern China and Korea, transmission is more prone to occur during autumn and winter (Kim & Jang, 2010; Liu et al., 2006). In addition, several attempts have been made to explore the climate‐epidemic association by using statistical models (Kim & Kim, 2018; Seto et al., 2017; Wei et al., 2017; Zheng et al., 2018). For example, Seto et al. (2017) adopted a negative binomial regression to examine the effects of climate factors on scrub typhus in the Yamagata Prefecture of Japan. Kim and Kim (2018) used a hierarchical Bayesian Poisson model to reveal a positive correlation between scrub typhus cases and precipitation in summer in South Korea. Wei et al. (2017) employed two quantitative models to identify the key roles that climate factors (i.e., precipitation and relative humidity) played in driving the seasonal activity of scrub typhus in Guangzhou. Zheng et al. (2018) analyzed the spatial heterogeneity of scrub typhus in southern China from 2007 to 2017 through a boosted regression tree modeling procedure with several spatial correlates and concluded that the transmission of the disease is highly dependent on temperature and relative humidity.

Although the scientific community has studied the climate‐epidemic association for decades at different temporal and spatial scales (Kim & Kim, 2018; Kim & Jang, 2010; Seto et al., 2017; Wei et al., 2017; Zheng et al., 2018), there has been little research on the climate impacts on spatiotemporal dynamics of scrub typhus, especially in the context of climate warming. Using scrub typhus in China as a case study, we combined national surveillance data of scrub typhus from 2010 to 2019 with a climate‐driven generalized additive mixed model to explore the quantitative linkages of climate factors and the spatiotemporal dynamics of the disease. We use this relationship to quantify the potential effect of climate change on future scrub typhus risk under various trajectories for greenhouse gas concentrations as representative concentration pathways (RCPs) for three future time periods (the 2030s, 2050s, and 2080s).

## 2 MATERIALS AND METHODS

### 2.1 Data

#### 2.1.1 Scrub typhus cases

Individual‐level data on human cases of scrub typhus in China spanning from 2010 to 2019 were obtained from the Chinese Center for Disease Control and Prevention (China CDC), including current address of patients, date of illness onset, and case classification (confirmed, clinically diagnosed, suspected). Individual‐level data on human cases had been de‐identified to protect patient confidentiality. Patients with the suspected classification were excluded from this study due to the uncertainty. All clinically diagnosed and laboratory‐confirmed human cases were included in our analysis, with a total number of 166,839 from 2010 to 2019. It was determined by the National Health Commission of China that the collection of data on scrub typhus cases was conducted as part of ongoing public health surveillance of infectious disease and was, therefore, exempt from institutional review board assessment.

#### 2.1.2 Terrain data

Previous studies have suggested the apparent influence of elevation on the distribution of small mammals and their ectoparasitic chigger mites. For example, there are many more species of small mammals and mites found in Mountainous land than in cultivated flatland in Yunnan, China (Peng et al., 2018). Additionally, Zheng et al. (2018) reveal that elevation is one of the associate factors for scrub typhus in southern China. Thus, we adopted elevation as a covariate into our model. The gridded terrain data were downloaded from the Data Center for Resources and Environmental Sciences, Chinese Academy of Science (RESDC) (http://www.resdc.cn), with a spatial resolution of 1 km × 1 km.

#### 2.1.3 Historical climate data

Several lines of evidence suggest that the abundance and activity of chigger mites are strongly associated with climate conditions (Gubler et al., 2001; Jenkins, 1948; Sasa, 1961), which determine the chance of acquiring scrub typhus. For example, a positive association was observed between the rate of larval movement and temperature (Jenkins, 1948). Another example is the moderate amount of precipitation favouring humidity in habitats and food availability of chiggers, as opposed to the possible damage done to chigger mite eggs or scrub typhus nests by heavy precipitation to some extent (Gubler et al., 2001). In addition, various studies have explored the relationship between climate factors and scrub typhus, illustrating that the spatial distribution of the disease is affected by climate (Zheng et al., 2018). For instance, a study conducted in Guangzhou of China stated that every one‐degree and one‐millimetre increase in temperature and precipitation corresponded to an increase of 15% and 0.1%, respectively, in the number of monthly scrub typhus cases (Tiegang Li et al., 2014). To explain the spatial–temporal patterns of scrub typhus in China, mean temperature (°C), relative humidity (%), and precipitation (mm) were selected as covariates for our model based on previous research. The daily climate records of weather stations in China on mean temperature (°C), precipitation (mm), and relative humidity (%) during 2010 to 2019 were collected from the China Meteorological Data Service Center (http://data.cma.cn), from which 1 km × 1 km gridded climate data were generated by ANUSPLIN‐SPLINA software.

#### 2.1.4 Climate change projection data

The Coupled Model Intercomparison Project Phase 5 (CMIP5) project is a collaboration among climate modelers to produce a consistent set of climate model outputs for various RCP emission scenarios (https://esgf‐node.llnl.gov/search/cmip5/; Taylor et al., 2012). To predict future spatial–temporal patterns of scrub typhus, we included the monthly projected climate data (precipitation, temperature, and relative humidity) simulated by four general climate models (GCMs) under various RCPs from data sets of CMIP5, with a spatial resolution of 0.5° × 0.5°, corresponding to about 50 km × 50 km grid cells at the equator. Motivated by previous studies (Caminade et al., 2014; Colon‐Gonzalez et al., 2018), we selected four CMIP5 models to sample a wide range of potential climate changes: Hadley Global Environment Model 2—Earth System (HadGEM2‐ES), Institute Pierre Simon Laplace Coupled Model Version Five A—Low Resolution (IPSL‐CM5ALR), Geophysical Fluid Dynamics Laboratory Earth System Model with MOM, version 4 component (GFDL‐ESM2M), and Model for Interdisciplinary Research on Climate (MIROC5). In addition, we chose projections for three greenhouse gas emission scenarios corresponding to three levels of radiative forcing (W/m^2^) by 2100, RCPs 4.5, 6.0, and 8.5. Specifically, RCP4.5 and RCP6.0 represent stabilized scenarios with different government interventions (i.e., employment in the technologies and strategies for reducing greenhouse gas emissions) by 2100 and corresponding mean global warming of around 2.4°C and 3.1°C, respectively, while RCP8.5 assumes a continuing rise in emissions in the absence of climate change mitigation policies with no stabilization and corresponding warming of around 4.9°C by 2100. For each of these three scenarios, gridded climate data were extracted as three 10‐year time slices: the 2030s (2030–2039), 2050s (2050–2059), and 2080s (2080–2089). Afterward, the projected daily climate data were aggregated to the month‐county level, regarded as the future climate variables to predict climate‐related scrub typhus situation in the 2030s, 2050s, and 2080s under various RCPs.

### 2.2 Analysis

#### 2.2.1 Model specification

The association between local climate conditions and scrub typhus cases was estimated by a general additive model with a quasi‐Poisson distribution (Equation 1).

(1) $$Y_{i,t}=\alpha +f\left(X_{i,t‐l}\right)+{\text{Elevation}}_{i}+{\text{Area}}_{i}+\varepsilon$$

The term α denotes the intercept; the dependent variable *Y*_*i,t*_ was the number of scrub typhus cases at county *i* in time *t* (monthly/2010–2019); *l* was lag months; Elevation_*i*_ represents the average altitude of county *i*; *ε* was defined as the error term; *X*_*i,t−l*_ indicate climatic predictors (precipitation, mean temperature, and relative humidity) for month *t*_*−*_ *l*; *f()* was smooth function defined by thin‐plate splines in this study. Considering the amount of time required for the mite lifecycle and the incubation period of this disease, lags of 0–3 months (*l* = {0,1,2,3}) were used to examine the delayed effects of climate factors on scrub typhus cases. We fitted the association between climate variables and scrub typhus cases under 0–3 month time lags. More specifically, we used the climate variables in the preceding 0–3 months to predict the number of cases in the current month. A term of fixed effect (Area_*i*_) was included to account for the effects of unknown or unavailable variables in the model. We tested with both province and city fixed effects in our study to explain differences in the baseline level among areas. In summary, a total of eight model formulae were fitted in our study (Table 1). The mgcv package of *R* was implemented for general additive model analyses. We used values of GCV, *R*, and deviance explanation as model evaluation criteria. A model with lower GCV, higher *R*, and deviance explanation was determined to be more predictive and hence selected.

**TABLE 1:** Formulae and performance of climate‐driven generalized additive mixed models. Based on the fixed effects of province (models 1–4) and city (models 5–8), the performances of models with 0‐month (models 1 and 5), 1‐month (models 2 and 6), 2‐month (models 3 and 7), and 3‐month (models 4 and 8) lags of climate conditions were quantified by values of the coefficient of determination (*R*), generalized cross validation (GCV) criterion and the proportion of deviation explained

Name	Model formula	*R*	GCV	Deviance explained (%)
Model 1	$Y_{i,t}=f\left(P_{i,t}\right)+f\left(T_{i,t}\right)+f\left({\mathrm{RH}}_{i,t}\right)+{\mathrm{DEM}}_{i}+{\text{Province}}_{i}+\varepsilon$	0.621	1.977	38.6
Model 2	$Y_{i,t}=f\left(P_{i,t-1}\right)+f\left(T_{i,t-1}\right)+f\left({\mathrm{RH}}_{i,t-1}\right)+{\mathrm{DEM}}_{i}+{\text{Province}}_{i}+\varepsilon$	0.675	1.750	45.6
Model 3	$Y_{i,t}=f\left(P_{i,t-2}\right)+f\left(T_{i,t-2}\right)+f\left({\mathrm{RH}}_{i,t-2}\right)+{\mathrm{DEM}}_{i}+{\text{Province}}_{i}+\varepsilon$	0.689	1.692	47.4
Model 4	$Y_{i,t}=f\left(P_{i,t-3}\right)+f\left(T_{i,t-3}\right)+f\left({\mathrm{RH}}_{i,t-3}\right)+{\mathrm{DEM}}_{i}+{\text{Province}}_{i}+\varepsilon$	0.650	1.858	42.3
Model 5	$Y_{i,t}=f\left(P_{i,t}\right)+f\left(T_{i,t}\right)+f\left({\mathrm{RH}}_{i,t}\right)+{\mathrm{DEM}}_{i}+{\text{City}}_{i}+\varepsilon$	0.750	1.412	56.2
Model 6	$Y_{i,t}=f\left(P_{i,t-1}\right)+f\left(T_{i,t-1}\right)+f\left({\mathrm{RH}}_{i,t-1}\right)+{\mathrm{DEM}}_{i}+{\text{City}}_{i}+\varepsilon$	0.791	1.205	62.6
Model 7	$Y_{i,t}=f\left(P_{i,t-2}\right)+f\left(T_{i,t-2}\right)+f\left({\mathrm{RH}}_{i,t-2}\right)+{\mathrm{DEM}}_{i}+{\text{City}}_{i}+\varepsilon$	0.802	1.149	64.3
Model 8	$Y_{i,t}=f\left(P_{i,t-3}\right)+f\left(T_{i,t-3}\right)+f\left({\mathrm{RH}}_{i,t-3}\right)+{\mathrm{DEM}}_{i}+{\text{City}}_{i}+\varepsilon$	0.773	1.298	59.7

The following steps of data manipulation and analyses were employed to fit the model. (a) Monthly aggregated scrub typhus cases were calculated from 2010 to 2019 at county level; (b) terrain and climate data were compiled from 2010 to 2019 as spatial covariates, which were extracted for each county using ArcGIS 10.6 software (ESRI Inc.); (c) the general additive model models described above were fitted based on assembled data to explore the relationship between climate covariates and scrub typhus cases, and the best‐fitted models were selected judging by their performance; (d) projected climate data were used under three RCPs from four GCM models to predict scrub typhus cases in mainland China in the 2030s, 2050s, and 2080s based on the best‐fitted model; (e) an ensemble approach was taken to average outputs from multi‐GCMs under future climate conditions.

#### 2.2.2 Seasonality of scrub typhus

To quantify the characteristics of scrub typhus seasonality, we calculated the duration time and peak month of scrub typhus case numbers. The duration time was defined as follows: the cumulative case numbers captured in the interval between the start and end accounted for >95% of the total cases throughout the epidemic. The peak month was defined as the month with the highest number of scrub typhus cases.

## 3 RESULTS

### 3.1 Historical spatiotemporal pattern of scrub typhus

**FIGURE 1:**
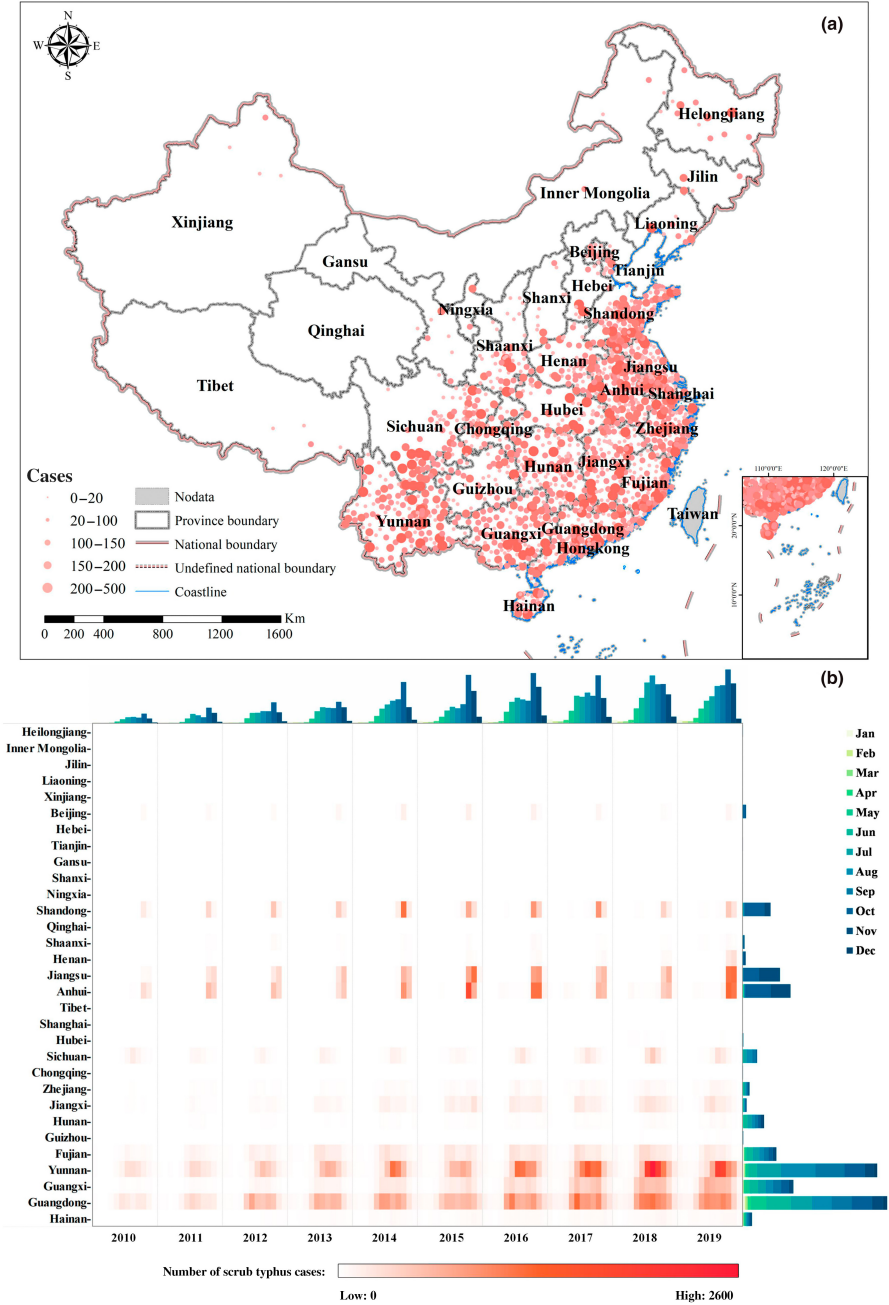
Spatiotemporal dynamics of reported scrub typhus cases in mainland China from 2010 to 2019. (a) Geographical distribution showing. Case numbers are distinguished by the size of the red circle. (b) Temporal variation showing. The main panel (heatmap) shows the monthly numbers of scrub typhus cases from 2010 to 2019 by province ordered by decreasing latitude, the top panel (cluster bar plot) shows the monthly national numbers of cases by time, and the right panel (stack bar plot) shows the total numbers of cases by province, consisting of cases in each month. Map lines delineate study areas and do not necessarily depict accepted national boundaries.

Our analysis comprised all clinically diagnosed and laboratory‐confirmed human scrub typhus cases from 2010 to 2019 with a total number of 166,839, affecting 31 provinces, 269 cities, and 1419 counties across mainland China, exhibiting a northward geographic expansion of scrub typhus during this period (Figure S1). In addition, the number of new cases per year increased steadily over time, from 4191 in 2010 to 27,438 in 2019 (Figure S2). We depict the spatiotemporal dynamic of scrub typhus in mainland China from 2010 to 2019 in Figure 1. Figure 1a illustrates the geographical distribution of scrub typhus cases at county level in mainland China during 2010–2019, with nearly all cases occurring in southern China. The most highly endemic areas were in provinces along the Southern and Eastern coast of China as well as Yunnan Province, while only sporadic cases were reported in northern provinces. The province with the most total reported cases was Guangdong, with 44,403 (26.6%), followed by Yunnan with 41,312 (24.7%). In addition, total case numbers in several southeast coastal provinces such as Jiangsu, Anhui, Fujian, and Guangxi exceeded 10,000. Information on the numbers of scrub typhus cases by province from 2010 to 2019 is presented in Table S1.

Figure 1b demonstrates the temporal variation in monthly scrub typhus cases in mainland China from 2010 to 2019, revealing a geographical divergence of scrub typhus seasonality across China. The peak season changes from summer to autumn as the latitude of the provinces increase, accompanied by a shortened epidemic duration. For instance, the epidemic season of this disease in Guangdong and Guangxi of Southern China lasted for 7 months, from May to November, with case numbers peaking in June. In Yunnan province, a peak in summer was observed in August, with the epidemic season extending from June to November. While in the northern regions (i.e., provinces of Jiangsu, Anhui, and Shandong), the maximum number of cases occurred in October, about 93% of which were concentrated in October and November. Nationally, a left‐skewed seasonal pattern of scrub typhus cases was observed during the same period. It started to soar in May and reached a peak in October before beginning to taper off in November (Figure S2), and 94% (159,604/166,839) of the cases were reported during May to November.

### 3.2 Climate driving factors on the spatiotemporal dynamics of scrub typhus

In this study, we build eight climate‐driven generalized additive mixed models to simulate the spatiotemporal dynamics of scrub typhus (see Section 2). The formulae and performance of these models are shown in Table 1. According to the results from Table 1, Model 3 and Model 7 had the best performances at their respective scales, with relatively higher deviance explained values and lower generalized cross validation (GCV) values, indicating that local climate conditions with a 2‐month lag provided the best fits compared with 0, 1, or 3‐month lags. The performance of models with city fixed effects (models 5–8) was generally better than those with provincial fixed effects (models 1–4) under the same climate conditions. Overall, Model 7 was the optimal choice for simulating the spatiotemporal dynamics of scrub typhus, with values for *R*, GCV, and deviance of 0.802%, 1.149%, and 64.3%, respectively.

**FIGURE 2:**
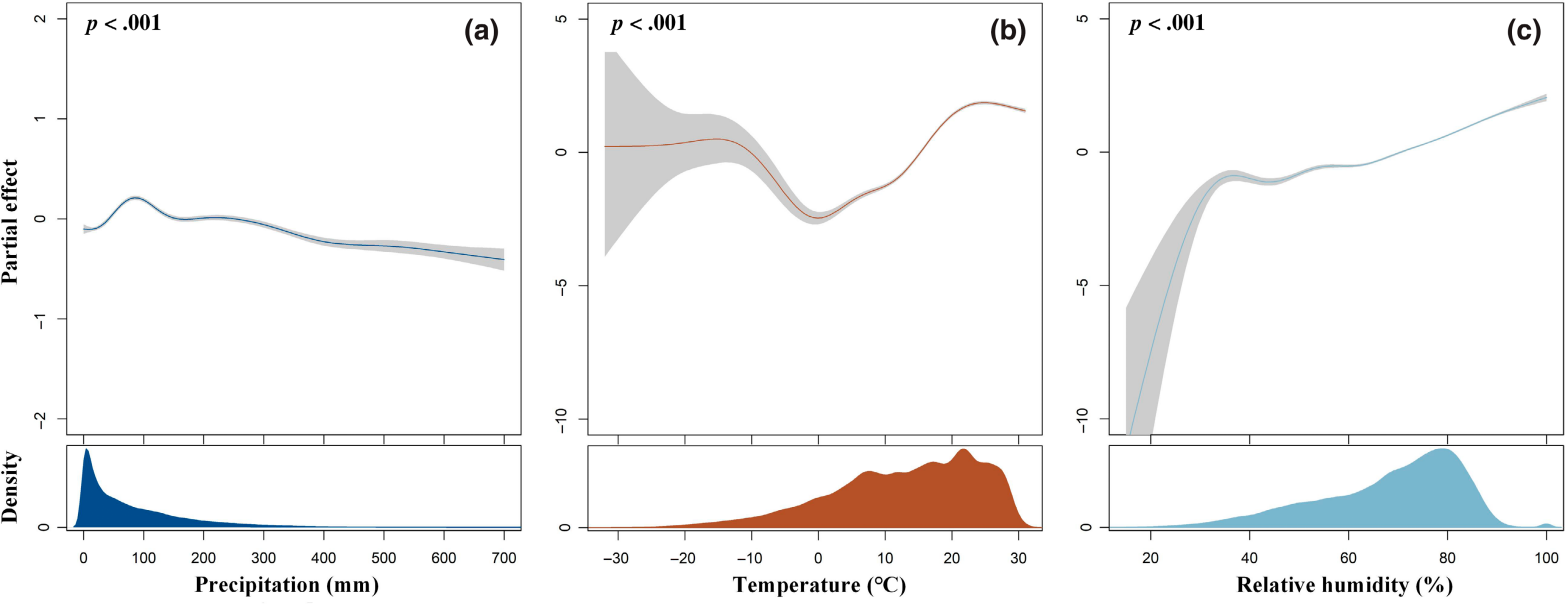
Potentially nonlinear influences of climate factors on scrub typhus cases in mainland China derived from model 7 (city fixed effect and 2‐month lagged) fitted from data covering from 2010 to 2019. (a) Precipitation (mm), (b) mean temperature (°C), and (c) relative humidity (%). The solid lines represent median estimates, and the shaded area means and corresponding 2.5th and 97.5th quantiles.

Significant associations were found between climate factors and the spatiotemporal dynamics of scrub typhus (Table S2). The climate‐disease nonlinear links are depicted in Figure 2 using the optimal model (model 7). There was an inverted U‐shaped relationship between precipitation and the number of scrub typhus cases (*F* = 90.55, *p* < .001) with a peak at nearly 100 mm/month and a negative correlation for precipitation above 100 mm/month. The relationship between mean temperature and the number of scrub typhus cases was complex (*i* = 2174.21, *p* < .001). For example, there was a significant negative correlation between mean temperature and the number of scrub typhus cases when the mean temperature was between −8 to 0°C, and a generally positive association when it was above 0°C. However, a slight decrease appears when mean temperatures reach 25°C. There was also a nonlinear but generally increasing association between relative humidity (*F* = 890.03, *p* < .001) and the 2‐month lagged number of scrub typhus cases. Additionally, the potential nonlinear effects of ambient climate factors on the numbers of scrub typhus cases across lags of 0–3 months at two different scales are presented in Figure S3.

### 3.3 Predicting scrub typhus dynamics under future climate changes

**FIGURE 3:**
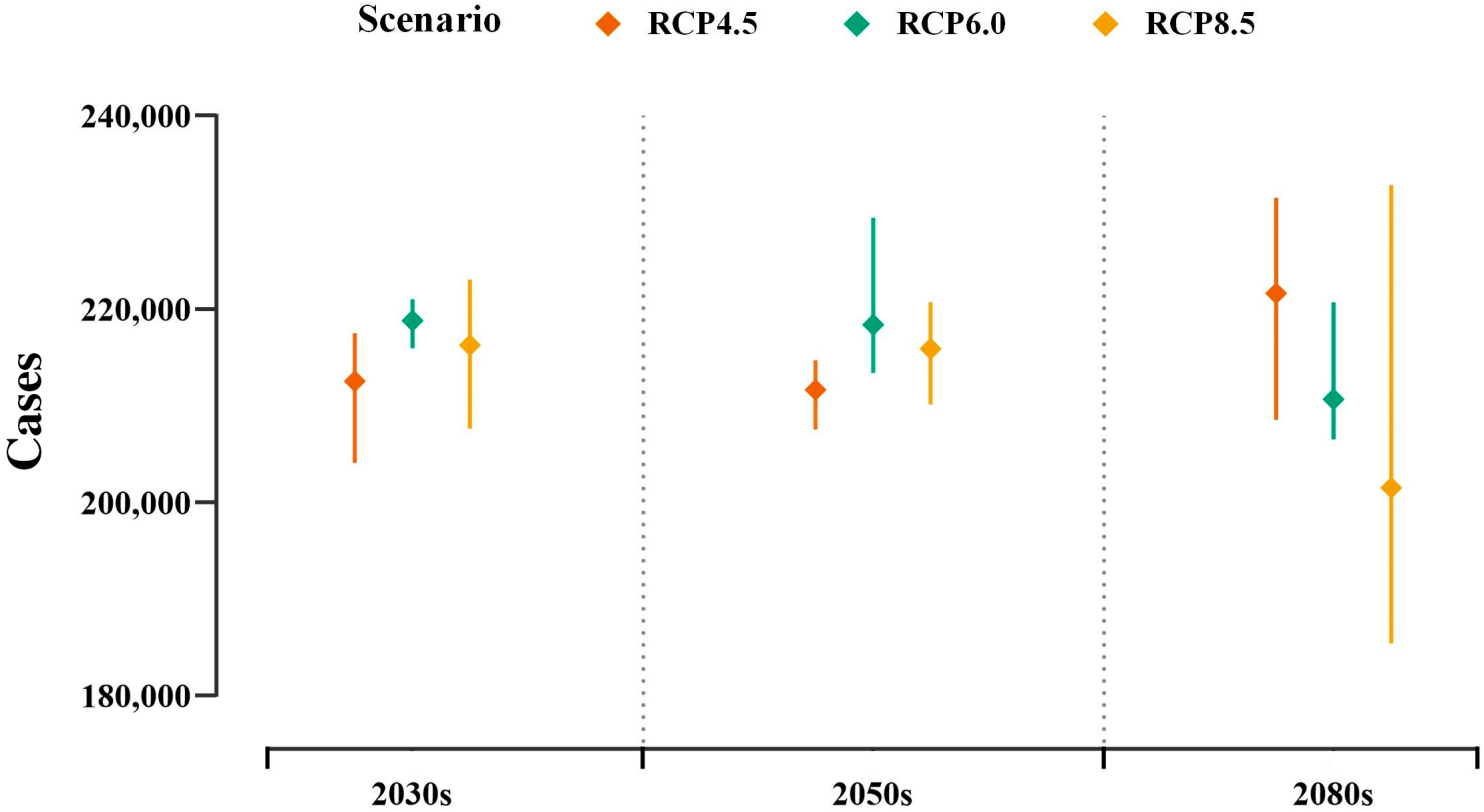
The multi‐GCM ensemble mean of the predicted number of national scrub typhus cases under different climate change scenarios (RCP4.5, RCP6.0, and RCP8.5) in the 2030s, 2050s, and 2080s. Rhombic points show the mean estimates of national scrub typhus cases and error bars are defined as the range.

We predicted the spatial–temporal patterns of scrub typhus in the 2030s, 2050s, and 2080s under various RCPs using the best‐fitting model (model 7) based on projected climate data derived from four GCMs (see Section 2). Figure 3 depicts the predicted total numbers of scrub typhus cases in mainland China under different RCPs, indicating that the projected number of scrub typhus cases is likely to peak in the 2080s under RCP4.5, increasing at 39% from baseline 2010s (Table S3). Under scenarios of RCP6.0 and RCP8.5, the predicted numbers of cases will peak in the 2050s, with an increase of 37% and 35% compared with the 2010s, respectively (Table S3).

**FIGURE 4:**
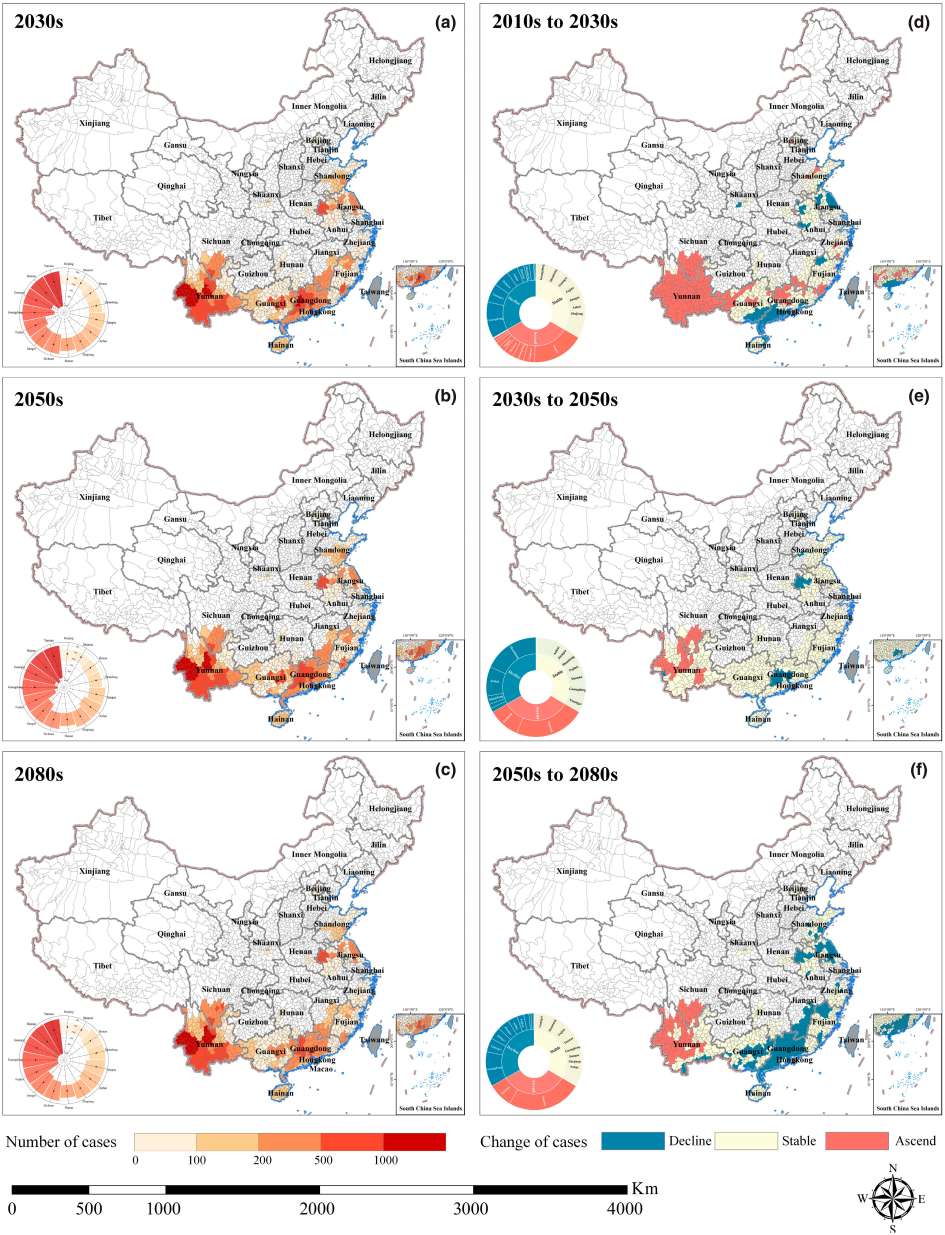
Spatiotemporal pattern of the predicted scrub typhus cases and its change over time under RCP6.0 scenario. The left panel depicts the spatial and temporal pattern of the four‐GCM ensemble mean of the predicted scrub typhus cases for the 2030s (a), 2050s (b), and 2080s (c). Radar‐Bar map denotes the seasonality characteristics of scrub typhus cases by province. The circumference is divided into 15 provinces in a clockwise direction, and the radius from inside to outside represents a particular month from January to December. Bar area represents the epidemic duration of scrub typhus, the black dot means the peak month, which defines as the month with the highest number of scrub typhus cases. The right panel shows changes in projected scrub typhus cases from the 2010s to 2030s (d), from the 2030s to 2050s (e), from the 2050s to 2080s (f), respectively. Colors determine different change types in cases number. Blue represents a decline, meaning that the number of cases decreased by more than 20. Red represents ascend, meaning that the number of cases increased by more than 20. Yellow represents stability, meaning that the number of cases fluctuated by no more than 20. Sunburst chart shows the proportion of provinces for each changes type. Map lines delineate study areas and do not necessarily depict accepted national boundaries.

Overall, we predicted minimal changes in the geographical patterns of predicted scrub typhus cases in mainland China, but significant regional changes in case numbers were expected between the 2010s and 2080s (Figure 4; Figures S4 and S5). Under the RCP6.0 scenario, the predicted geographical distribution of scrub typhus cases in the 2030s, 2050s, and 2080s are presented in Figure 4a–c, respectively. The projected distribution is concentrated in southern and eastern China. Three observed epidemiologic cluster regions are (1) Yunnan province, (2) Guangdong and Guangxi provinces, and (3) Shandong, Anhui, and Jiangsu provinces, accounting for about 44%, 25%, and 15% of total projected cases, respectively (Table S4). Changes in cases number differed among regions (Figure 4d–f), where the most marked increase in cases are predicted in Yunnan province and in southern Sichuan by the 2080s. At the same time, some areas in Guangdong, Guangxi, Jiangxi, and parts of Jiangsu and Anhui are predicted to see declining cases number, but other areas are predicted to remain stable by the 2080s. Compared with the RCP6.0 scenario, there are almost no areas where we predicted a decline in cases number by the 2080s under RCP4.5 (Figure S4), while more areas are likely to see the decline in cases number by the 2080s under RCP8.5 (Figure S5). In addition, we also quantified the model uncertainty in spatial predictions for scrub typhus based on the coefficient of variation values calculated for each county across the model ensemble (Figure S6). The uncertainty analysis reveals low prediction uncertainty in most epidemic areas.

The estimated seasonality profiles for each province in the future under RCP6.0 present a generally extended epidemic duration compared with the 2010s. The epidemic season in northern China was predicted to extend in the next few decades compared to that in the 2010s. For example, the predicted seasonality in Shandong in the 2080s lasts from July to December, 1 month longer compared with its historical epidemic duration (from July to November). In addition, the scrub typhus cases tend to peak earlier in southern China. For instance, the month when the case numbers peak in Guangxi and Guangdong changed from August in the 2010s to July in the 2080s.

## 4 DISCUSSION

Our study builds on previous research that has linked spatiotemporal dynamics of scrub typhus to climate variables (Kim & Jang, 2010; Seto et al., 2017) and makes further progress by explaining the disease dynamics and quantifying the climate‐change impacts under various RCPs in three future time periods based on a climate‐driven generalized additive mixed model and several GCMs. For this study, we compile contemporary scrub typhus monthly reports for mainland China at county level based on a nationwide longitudinal disease surveillance dataset spanning 10 years from China CDC. Such an assembled set of spatiotemporal scrub typhus data paired with climate variables allowed us to model the dynamics of the disease transmission in more detail than in previous research (Wei et al., 2017; Yang et al., 2014; Zheng et al., 2018). Based on the comprehensive dataset, we found that temperature, precipitation, and relative humidity have complex nonlinear influences on the seasonal epidemic of scrub typhus with a delay of 2 months through exploring the relationship between climate factors and scrub typhus dynamics using a general additive model modelling framework.

These relationships could be explained by the complex interactions at the environment–vector–human interface. On the one hand, climate variables are certainly important factors influencing the development of mite larvae (the main vector of scrub typhus; Elliott et al., 2019). On the other hand, climate conditions have also been documented to mitigate the exposure to vectors by affecting the frequency of outdoor activities (Marks et al., 1996). More specifically, when the temperature drops too low or rises too high, it will be difficult for chiggers to feed with reduced opportunity for attaching to humans (Elliott et al., 2019; Moniuszko & Mąkol, 2016), while a temperate climate may trigger the development of eggs into larvae and thus increases exposure risk (Jameson Jr, 1967). Warmth could also enhance farming and recreational activities that contribute to high‐risk exposure (Yao et al., 2019). Although the inverse U‐shaped relationship between precipitation and the disease risk differs from previous studies (Chen et al., 2016; Yang et al., 2014), it may be explained by two opposing effects: precipitation increases the population of the larvae while reducing human outdoor activities. Humid environments are beneficial to the reproduction of most chigger mites, which may result in a positive association between relative humidity and the spread of scrub typhus (Lv et al., 2020). It is also important to note that the 2‐month lagged effect may be due to the sum of the duration of the larvae development and the incubation period of the pathogen, which is nearly 2 months (Newton & Day, 2020; Shatrov & Kudryashova, 2006).

Previous studies have suggested that the future spatiotemporal distribution of vector‐borne disease can be predicted using either a mechanistic model or a statistical model (Altizer et al., 2013; Gething et al., 2010; Messina et al., 2019; Morin & Comrie, 2013; Ryan et al., 2021). Compared with mechanistic modeling approaches that are highly dependent on the well‐identified and temperature‐based biological processes related to vector‐borne disease spread (Messina et al., 2015), which ignore the effects of precipitation and relative humidity, statistical modeling approaches can better fit the empirical relationship observed from the more comprehensive historical data to predict the future risk. There are several studies about predicting the potential risk of scrub typhus across different geographic locations (Xin et al., 2020; Yao et al., 2019; Zheng et al., 2018). However, such predictions derived from yearly mean average values of covariates that only represented a long‐term average distribution of the disease risk and could not infer seasonal patterns of scrub typhus. By contrast, our analysis goes beyond previous studies regarding the projection of the scrub typhus spread risk by revealing the future spatiotemporal dynamics of the disease. Overall, our findings show that nationally, the projected scrub typhus cases will increase in the future under different RCPs compared with the 2010s. Meanwhile, the future spatiotemporal response of scrub typhus to climate change across China is observed to be heterogeneous, which is likely due to specific combinations of regional climate conditions in future climate scenarios. For example, under RCP6.0 scenario, future climate change will extend the duration of the scrub typhus seasonality in northern China while the case number tends to peak earlier in southern China. As to its influence on spatial variation of cases, that is, it is highly possible to see the biggest increase in scrub typhus cases in Yunnan province, while case number in some other regions, including southern Guangdong and Guangxi, tend to decrease in the future. This change is, to some extent, varying under different climate change scenarios (Figure 4; Figures S4 and S5).

It should be noted that there are some limitations that need to be considered. First, scrub typhus datasets were collected from the national disease surveillance system where the data quality may be influenced by the key steps in surveillance, including the availability of health facilities and laboratory diagnostics, under reporting, and completeness and accuracy of data over the years (Li et al., 2020). A generalized additive mixed model was employed to reduce these effects, but the bias in data may, to some degree, add uncertainty to our results. Second, our quantitative analysis was based on the largest contemporary and most reliable scrub typhus datasets yet assembled. Still, since there are no publicly available datasets of the factors (i.e., landscape change, urbanization, socioeconomic conditions, human behavior patterns, and public health policy) (Yao et al., 2019; Zheng et al., 2018) associated with the disease for the future periods, these factors are therefore not included in our analysis. Thirdly, the absence of field or laboratory experiments to verify the empirical relationships derived from the historical data signifies the need for future research about the impacts of climate on scrub typhus. In the aggregate, our results provide a better understanding of how climate factors affect the spatiotemporal dynamics of scrub typhus and quantify the potential impacts of climate change on this disease.

## AUTHOR CONTRIBUTIONS

Fangyu Ding, Dong Jiang, Canjun Zheng, and Tian Ma conceived and designed the study. Tian Ma, Fangyu Ding, and Qian Wang collected the data and carried out the computations. Fangyu Ding, Tian Ma, Qian Wang, Canjun Zheng, and Dong Jiang analyzed the data. Fangyu Ding, and Tian Ma wrote the paper. Qian Wang, Dong Jiang, Canjun Zheng, Mengmeng Hao, Richard James Maude, Nicholas Philip John Day, Shengjie Lai, Shuai Chen, and Liqun Fang gave some useful suggestions to this work. All authors critically reviewed the manuscript and approved the final manuscript.

## CONFLICT OF INTEREST

The authors declare no competing interests.

## DATA AVIALABILITY STATEMENT

The derived data that support the results and figures in this studyare openly available in Dryad (https://doi.org/10.5061/dryad.x69p8czmz).

## Supporting information


Appendix S1
