# Supplementary material for: Climate drives the spatiotemporal dynamics of scrub typhus in China
Source: Glob Chang Biol. 2022 Sep 2;28(22):6618–28. doi: 10.1111/gcb.16395 (PMC9825873; doi:10.1111/gcb.16395)
Supplement: Supplementary file 1 — Appendix S1 [file GCB-28-6618-s001.docx]

**Supplementary Information**

For

**Climate drives the** **spatiotemporal dynamics of scrub typhus in China**

Fangyu Ding^1,2^, Qian Wang^3,4^, Mengmeng Hao^1,2^, Richard James Maude^3,4,5^, Nicholas Philip John Day^3,4^, Shengjie Lai^6^, Shuai Chen^1,2^, Liqun Fang^7^, Tian Ma^1,2,*^, Canjun Zheng^8,*^, Dong Jiang^1,2,*^

^1^Institute of Geographic Sciences and Natural Resources Research, Chinese Academy of Sciences, Beijing 100101, China.

^2^College of Resources and Environment, University of Chinese Academy of Sciences, Beijing 100049, China.

^3^Centre for Tropical Medicine and Global Health, Nuffield Department of Medicine, University of Oxford, Oxford, United Kingdom.

^4^Mahidol Oxford Tropical Medicine Research Unit, Faculty of Tropical Medicine, Mahidol University, Bangkok, Thailand.

^5^Harvard TH Chan School of Public Health, Harvard University, Boston, USA.

^6^WorldPop, School of Geography and Environmental Science, University of Southampton, Southampton, UK.

^7^State Key Laboratory of Pathogen and Biosecurity, Beijing Institute of Microbiology and Epidemiology, Beijing, China.

^8^Chinese Center for Disease Control and Prevention, Beijing, China.

These authors contributed equally: Fangyu Ding, Qian Wang.

^*^ Correspondence and requests for materials should be addressed to: Tian Ma (mat.19b@igsnrr.ac.cn), Canjun Zheng (zhengcj@chinacdc.cn) and Dong Jiang (jiangd@igsnrr.ac.cn).

**Contents of this file:**

Figures S1 to S6

Tables S1 to S4

## Supplementary Figures

**
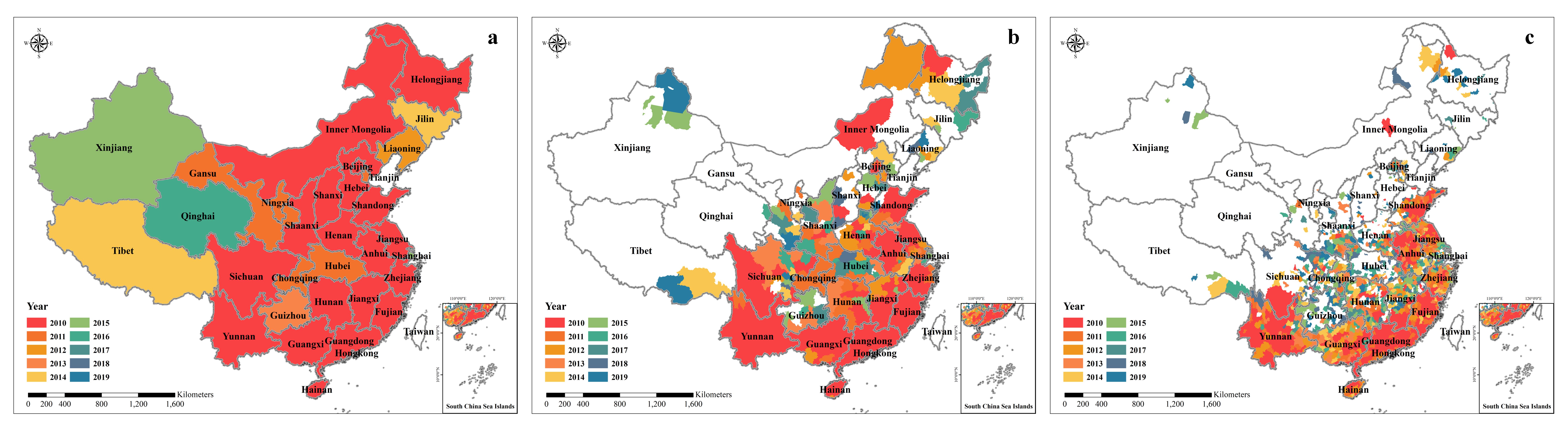
Figure S1. The spatial dynamics of scrub typhus by (a) province, (b) city, and (c) county in mainland China from 2010 to 2019.** Colors represent the first year in this range in which cases were reported.


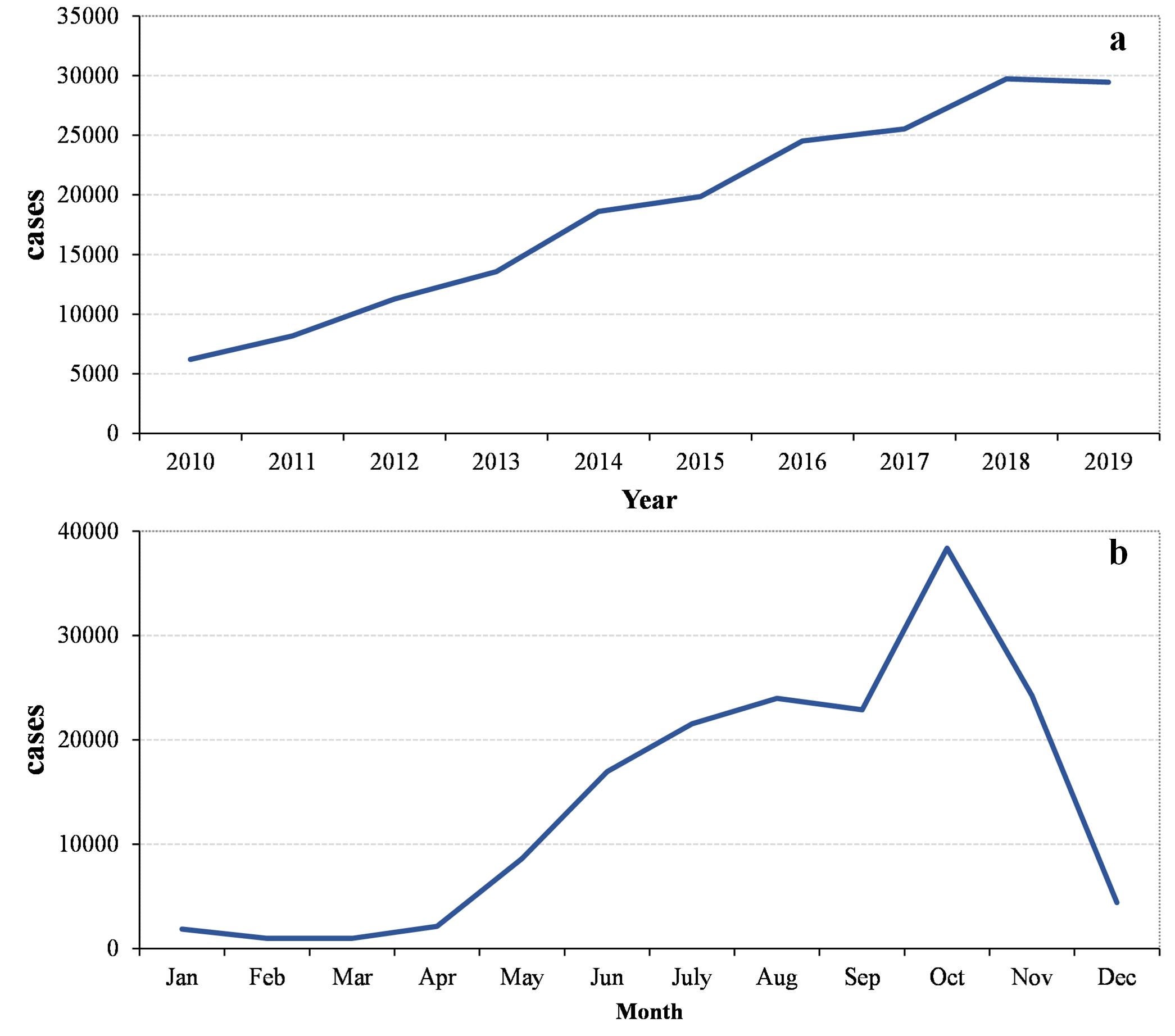


**Figure S2. The number of scrub typhus cases in mainland China from 2010 to 2019 by year (a) and month (b).**


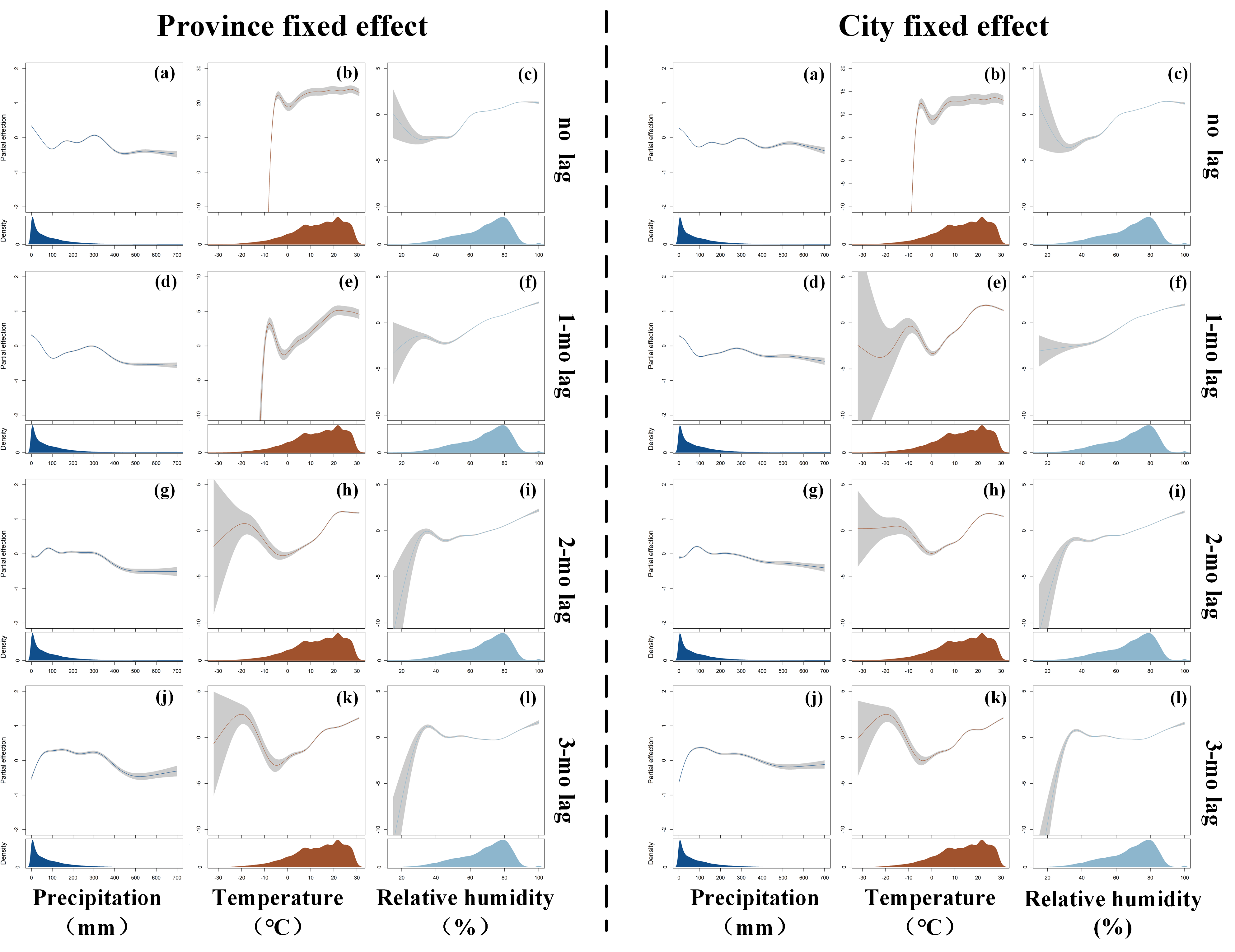


**Figure S3. Relationship curves between scrub typhus cases and climate variables for 8 models.** Left panel Models 1-4, right panel Models 5-8. The solid lines represent median estimates, and the shaded area means corresponding 2.5th and 97.5th quantiles.


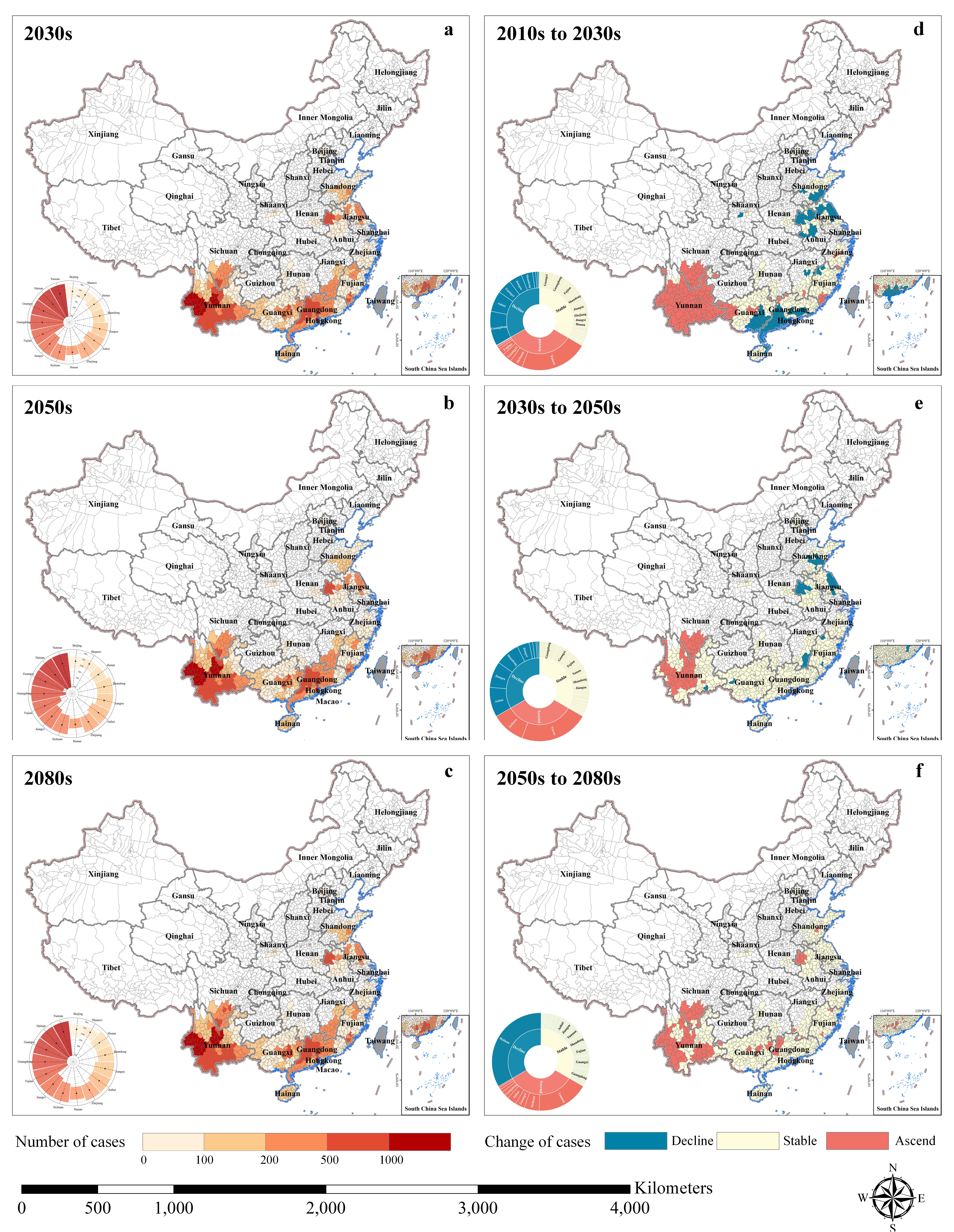


**Figure S4. Spatio-temporal pattern of the predicted scrub typhus cases and its change over time under RCP4.5 scenario.** The left panel depicts the spatial and temporal pattern of the four-GCM ensemble mean of the predicted scrub typhus cases for 2030s (a), 2050s (b), and 2080s (c). Radar-bar map denotes the seasonality characteristics of scrub typhus cases by province. The circumference is divided into 15 provinces in a clockwise direction, and the radius from inside to outside represents a particular month from January to December. Bar area represents the epidemic duration of scrub typhus, the black dot means the peak month, which defines as the month with the highest number of scrub typhus cases. The right panel shows changes in projected scrub typhus cases from 2010s to 2030s (d), from 2030s to 2050s (e), from 2050s to 2080s (f) respectively. Colors determine different change types in cases number. Blue represents a decline, meaning that the number of cases decreased by more than 20. Red represents ascend, meaning that the number of cases increased by more than 20. Yellow represents stability, meaning that the number of cases fluctuated by no more than 20. Sunburst Chart shows the proportion of provinces for each changes type.


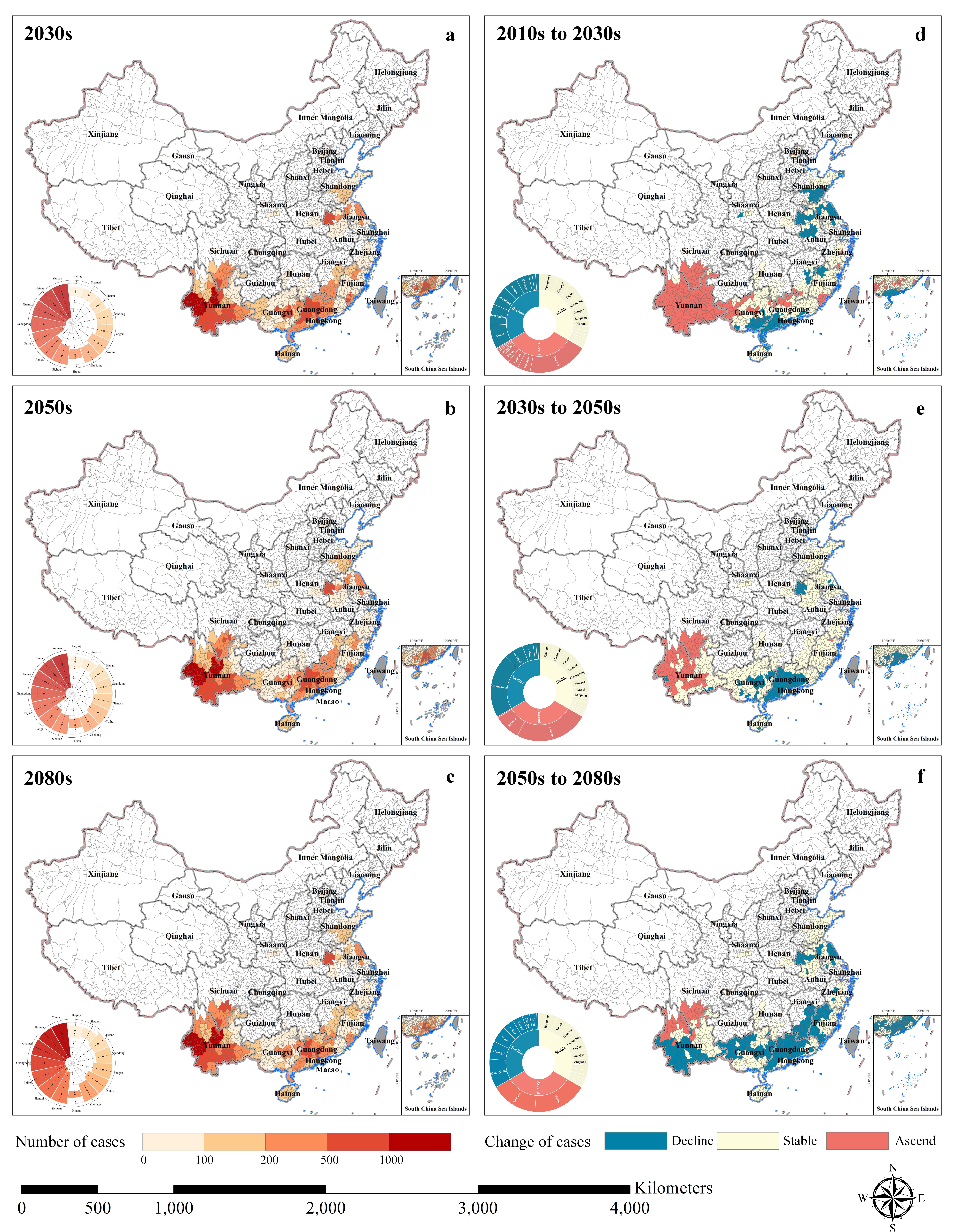


**Figure S5.** **Spatio-temporal pattern of the predicted scrub typhus cases and its change over time under RCP8.5 scenario.** The left panel depicts the spatial and temporal pattern of the four-GCM ensemble mean of the predicted scrub typhus cases for 2030s (a), 2050s (b), and 2080s (c). Radar-bar map denotes the seasonality characteristics of scrub typhus cases by province. The circumference is divided into 15 provinces in a clockwise direction, and the radius from inside to outside represents a particular month from January to December. Bar area represents the epidemic duration of scrub typhus, the black dot means the peak month, which defines as the month with the highest number of scrub typhus cases. The right panel shows changes in projected scrub typhus cases from 2010s to 2030s (d), from 2030s to 2050s (e), from 2050s to 2080s (f) respectively. Colors determine different change types in cases number. Blue represents a decline, meaning that the number of cases decreased by more than 20. Red represents ascend, meaning that the number of cases increased by more than 20. Yellow represents stability, meaning that the number of cases fluctuated by no more than 20. Sunburst Chart shows the proportion of provinces for each changes type.


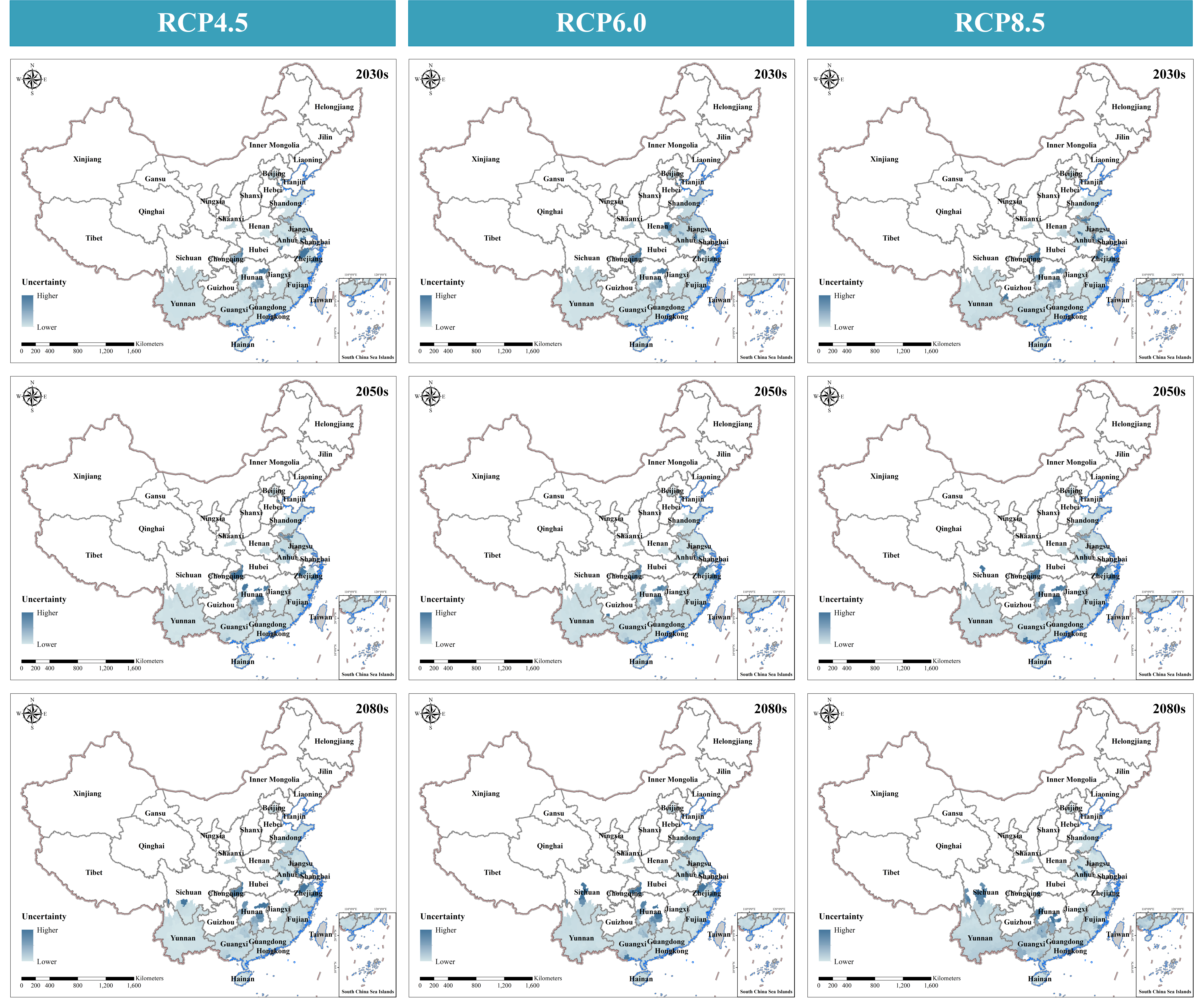


**Figure S6. The model uncertainty of spatial predictions for scrub typhus under different RCPs.**

## Supplementary Tables

**Table S1. Numbers of Scrub typhus case by province from 2010 to 2019.**

| Province | Year | | | | | | | | | | Total |
| --- | --- | --- | --- | --- | --- | --- | --- | --- | --- | --- | --- |
|  | 2010 | 2011 | 2012 | 2013 | 2014 | 2015 | 2016 | 2017 | 2018 | 2019 |  |
| Beijing | 56 | 71 | 55 | 87 | 149 | 169 | 162 | 102 | 54 | 118 | 1023 |
| Tianjin | 0 | 3 | 4 | 16 | 7 | 1 | 1 | 5 | 3 | 7 | 47 |
| Hebei | 1 | 0 | 2 | 0 | 2 | 3 | 1 | 4 | 2 | 8 | 23 |
| Shanxi | 1 | 0 | 4 | 0 | 0 | 0 | 0 | 0 | 3 | 4 | 12 |
| Inner Mongolia | 1 | 0 | 1 | 3 | 1 | 0 | 1 | 0 | 1 | 0 | 8 |
| Total North | 59 | 74 | 66 | 106 | 159 | 173 | 165 | 111 | 63 | 137 | 1113 |
| Liaoning | 0 | 0 | 1 | 0 | 1 | 3 | 4 | 7 | 7 | 7 | 30 |
| Jilin | 0 | 0 | 0 | 0 | 1 | 1 | 1 | 1 | 0 | 0 | 4 |
| Heilongjiang | 1 | 0 | 3 | 1 | 10 | 6 | 3 | 6 | 3 | 12 | 45 |
| Total Northeast | 1 | 0 | 4 | 1 | 12 | 10 | 8 | 14 | 10 | 19 | 79 |
| Shanghai | 0 | 0 | 0 | 0 | 0 | 1 | 0 | 0 | 1 | 0 | 2 |
| Jiangsu | 107 | 535 | 536 | 736 | 1150 | 2016 | 1767 | 1098 | 1107 | 2442 | 11494 |
| Zhejiang | 38 | 95 | 89 | 99 | 220 | 249 | 305 | 301 | 339 | 387 | 2122 |
| Anhui | 499 | 980 | 885 | 741 | 1449 | 2469 | 2559 | 1351 | 1176 | 2602 | 14711 |
| Fujian | 316 | 483 | 722 | 1091 | 1138 | 1211 | 1431 | 1301 | 1662 | 997 | 10352 |
| Jiangxi | 55 | 132 | 245 | 420 | 586 | 960 | 968 | 892 | 1281 | 1045 | 6584 |
| Shandong | 253 | 458 | 629 | 649 | 1535 | 1119 | 1336 | 1208 | 593 | 776 | 8556 |
| Total East | 1268 | 2683 | 3106 | 3736 | 6078 | 8025 | 8366 | 6151 | 6159 | 8249 | 53821 |
| Henan | 7 | 17 | 10 | 21 | 36 | 64 | 89 | 75 | 112 | 495 | 926 |
| Hubei | 0 | 3 | 2 | 8 | 10 | 7 | 26 | 33 | 67 | 37 | 193 |
| Hunan | 24 | 75 | 52 | 61 | 123 | 137 | 157 | 167 | 222 | 214 | 1232 |
| Total Central | 31 | 95 | 64 | 90 | 169 | 208 | 272 | 275 | 401 | 746 | 2351 |
| Guangdong | 1143 | 1527 | 3386 | 3780 | 4529 | 4374 | 5703 | 6463 | 7078 | 6420 | 44403 |
| Guangxi | 128 | 178 | 397 | 560 | 1129 | 1233 | 1775 | 2935 | 3490 | 3743 | 15568 |
| Hainan | 14 | 33 | 96 | 280 | 337 | 341 | 424 | 445 | 462 | 462 | 2894 |
| Total South | 1285 | 1738 | 3879 | 4620 | 5995 | 5948 | 7902 | 9843 | 11030 | 10625 | 62865 |
| Chongqing | 0 | 2 | 1 | 5 | 7 | 7 | 15 | 8 | 12 | 13 | 70 |
| Sichuan | 372 | 219 | 215 | 303 | 339 | 253 | 518 | 628 | 984 | 631 | 4462 |
| Guizhou | 2 | 0 | 0 | 6 | 3 | 18 | 14 | 32 | 44 | 67 | 184 |
| Yunnan | 1159 | 1322 | 1861 | 2657 | 3804 | 3146 | 5175 | 6412 | 8950 | 6826 | 41312 |
| Tibet | 0 | 0 | 0 | 0 | 1 | 1 | 3 | 0 | 1 | 2 | 8 |
| Total Southwest | 1531 | 1543 | 2077 | 2971 | 4154 | 3425 | 5725 | 7080 | 9991 | 7539 | 46036 |
| Shaanxi | 16 | 35 | 76 | 45 | 41 | 48 | 80 | 45 | 49 | 117 | 552 |
| Gansu | 0 | 3 | 0 | 0 | 2 | 1 | 0 | 3 | 0 | 5 | 14 |
| Qinghai | 0 | 0 | 0 | 0 | 0 | 0 | 1 | 0 | 0 | 0 | 1 |
| Ningxia | 0 | 1 | 0 | 0 | 2 | 0 | 0 | 0 | 0 | 0 | 3 |
| Xinjiang | 0 | 0 | 0 | 0 | 0 | 2 | 0 | 0 | 1 | 1 | 4 |
| Total Northwest | 16 | 39 | 76 | 45 | 45 | 51 | 81 | 48 | 50 | 123 | 574 |
| Total whole country | 4191 | 6172 | 9272 | 11569 | 16612 | 17840 | 22519 | 23522 | 27704 | 27438 | 166839 |

Note: Rows in grey color represents provinces with cumulative number of cases < 100 in 2010 - 2019.

**Table S2. Summary statistics of results of Models 1 to 8.**

| Variable | Model 1 | | Model 2 | | Model 3 | | Model 4 | |
| --- | --- | --- | --- | --- | --- | --- | --- | --- |
|  | Estimate | p-value | Estimate | p-value | Estimate | p-value | Estimate | p-value |
| Intercept | -23.748 | <2e-16*** | -4.812 | <2e-16*** | -1.776 | <2e-16*** | -1.468 | <2e-16*** |
| Elevation | 1.273e-04 | <2e-16*** | 5.386e-04 | <2e-16*** | 6.475e-04 | <2e-16*** | 2.436e-04 | <2e-16*** |
|  | F value |  | F value |  | F value |  | F value |  |
| Precipitation | 459.2 | <2e-16*** | 493.1 | <2e-16*** | 77.39 | <2e-16*** | 106.8 | <2e-16*** |
| Temperature | 1552 | <2e-16*** | 4448.7 | <2e-16*** | 1667.18 | <2e-16*** | 794.9 | <2e-16*** |
| Relative humidity | 1693.6 | <2e-16*** | 2072.4 | <2e-16*** | 696.57 | <2e-16*** | 348.6 | <2e-16*** |
| Variable | Model 5 | | Model 6 | | Model 7 | | Model 8 | |
|  | Estimate | p-value | Estimate | p-value | Estimate | p-value | Estimate | p-value |
| Intercept | -13.375 | <2e-16*** | -1.498 | <2e-16*** | -1.479 | <2e-16*** | -1.290 | <2e-16*** |
| Elevation | 2.127e-04 | <2e-16*** | 5.008e-04 | <2e-16*** | 6.506e-04 | <2e-16*** | 4.713e-04 | <2e-16*** |
|  | F value |  | F value |  | F value |  | F value |  |
| Precipitation | 277.5 | <2e-16*** | 217 | <2e-16*** | 90.55 | <2e-16*** | 192.9 | <2e-16*** |
| Temperature | 113.6 | <2e-16*** | 2488 | <2e-16*** | 2174.21 | <2e-16*** | 1345.5 | <2e-16*** |
| Relative humidity | 1796.8 | <2e-16*** | 1467 | <2e-16*** | 890.03 | <2e-16*** | 461.1 | <2e-16*** |

Note: corresponding P values (***, P < 0.001; **, 0.001 < P < 0.01; *, 0.01 < P < 0.05).

**Table S3. Multi-GCMs ensemble mean (and range) of the predicted number of scrub typhus cases (thousands) in mainland China under different RCP scenarios.**

| **Scenario** | **Baseline** | **2030s** | **2050s** | **2080s** |
| --- | --- | --- | --- | --- |
| RCP 4.5 | 160 | 212 (204-217) | 212 (207-215) | 222 (208-231) |
| RCP 6.0 | 160 | 219 (216-221) | 219 (213-229) | 211 (206-220) |
| RCP 8.5 | 160 | 216 (207-223) | 216 (210-220) | 201(185 - 233) |

**Table S4. The estimated numbers of scrub typhus case by province under future climate projections, and the differences in predicted case numbers compared with the baseline 2010s.**

| **Province** | **RCP 4.5** | | | **RCP 6.0** | | | **RCP 8.5** | | |
| --- | --- | --- | --- | --- | --- | --- | --- | --- | --- |
|  | **2030s** | **2050s** | **2080s** | **2030s** | **2050s** | **2080s** | **2030s** | **2050s** | **2080s** |
| Beijing | 1007(87) | 1068(148) | 1041(121) | 1122(202) | 1075(155) | 1084(164) | 1018(98) | 1057(137) | 936(16) |
| Jiangsu | 8144(-2341) | 7526(-2959) | 7895(-2590) | 9019(-1466) | 9024(-1461) | 7076(-3409) | 8031(-2454) | 7761(-2724) | 6379(-4106) |
| Zhejiang | 1970(387) | 1816(233) | 1898(315) | 1920(337) | 2014(431) | 1645(62) | 1832(249) | 1887(304) | 1471(-112) |
| Anhui | 11521(-2606) | 10241(-3886) | 11110(-3017) | 13467(-660) | 12420(-1707) | 10043(-4084) | 11273(-2854) | 10746(-3381) | 9205(-4922) |
| Fujian | 10418(775) | 9884(241) | 10392(749) | 10671(1028) | 10362(719) | 9466(-177) | 10265(622) | 10118(475) | 8619(-1024) |
| Jiangxi | 6153(26) | 5715(-412) | 6133(6) | 6332(205) | 6269(142) | 5423(-704) | 6088(-39) | 6079(-48) | 4795(-1332) |
| Shandong | 7213(-854) | 6364(-1703) | 7023(-1044) | 8039(-28) | 7743(-324) | 6933(-1134) | 6688(-1379) | 6768(-1299) | 6458(-1609) |
| Henan | 667(-31) | 618(-80) | 653(-45) | 789(91) | 733(35) | 634(-64) | 689(-9) | 675(-23) | 583(-115) |
| Hunan | 236(-105) | 182(-159) | 233(-108) | 351(10) | 331(-10) | 136(-205) | 333(-8) | 142(-199) | 30(-311) |
| Guangdong | 39977(-4404) | 39284(-5097) | 39668(-4713) | 42182(-2199) | 40984(-3397) | 35675(-8706) | 41716(-2665) | 38092(-6289) | 32737(-11644) |
| Guangxi | 15106(-2) | 14460(-648) | 15177(69) | 15822(714) | 15772(664) | 13462(-1646) | 15774(666) | 13831(-1277) | 11300(-3808) |
| Hainan | 2564(-615) | 2567(-612) | 2489(-690) | 2535(-644) | 2530(-649) | 2256(-923) | 2618(-561) | 2337(-842) | 2178(-1001) |
| Sichuan | 7922(3742) | 8719(4539) | 9581(5401) | 7844(3664) | 8445(4265) | 9715(5535) | 8236(4056) | 9734(5554) | 11384(7204) |
| Yunnan | 96745(56180) | 100323(59758) | 105694(65129) | 95640(55075) | 98022(57457) | 104564(63999) | 98804(58239) | 103912(63347) | 102117(61552) |
| Shaanxi | 371(371) | 387(387) | 391(391) | 442(442) | 419(419) | 349(349) | 374(374) | 446(446) | 366(366) |
